# Physiological Effects of Touching the Wood of Hinoki Cypress (*Chamaecyparis obtusa*) with the Soles of the Feet

**DOI:** 10.3390/ijerph15102135

**Published:** 2018-09-28

**Authors:** Harumi Ikei, Chorong Song, Yoshifumi Miyazaki

**Affiliations:** 1 Department of Wood Engineering, Forestry and Forest Products Research Institute, 1 Matsunosato, Tsukuba, Ibaraki 305-8687, Japan; ikei0224@ffpri.affrc.go.jp; 2 Center for Environment, Health and Field Sciences, Chiba University, 6-2-1 Kashiwa-no-ha, Kashiwa, Chiba 277-0882, Japan; crsong1028@chiba-u.jp

**Keywords:** autonomic nervous activity, flooring wood, habitability, heart rate variability, near-infrared spectroscopy, physiological relaxation, prefrontal cortex activity, semantic differential method

## Abstract

We clarified the physiological effects of tactile stimulation of the soles of the feet with the wood of the Hinoki cypress (*Chamaecyparis obtusa*) based on measurements of prefrontal cortex and autonomic nervous activities. Nineteen female university-attending students (age: 21.2 ± 0.3 years) were included. Oxy-hemoglobin (oxy-Hb) concentrations in the prefrontal cortex were determined by using near-infrared time-resolved spectroscopy. The high frequency (HF) indicating parasympathetic nervous activity and the ratio of low frequency (LF)/HF indicating sympathetic nervous activity were measured using heart rate variability. To evaluate the psychological effects caused by contact with the materials, the modified semantic differential method was used. The soles of the participants’ feet were touched to a 600 × 600-mm plate made of Hinoki, which was finished in non-coating and brushing for 90 s. A marble plate served as the control. Next, subjective evaluation tests were administered to the participants. Compared with touching marble, touching Hinoki significantly (1) decreased the oxy-Hb concentrations in the left and right prefrontal cortices, which indicates decreased prefrontal cortex activity, (2) increased ln(HF), which indicates increased parasympathetic nervous activity, (3) decreased ln(LF/HF) ratio, which indicates decreased sympathetic nervous activity. Additionally, (4) according to subjective evaluations, the participants perceived themselves as being more “comfortable,” “relaxed,” “natural,” “warm,” “uneven,” “dry,” and “soft” after touching Hinoki. Thus, our cumulative findings indicate that touching Hinoki with the soles of the feet induces physiological relaxation.

## 1. Introduction

The urban environment causes “stress” among modern people. In 1950, less than one-third (30%) of the world’s population lived in urban settlements. In contrast, in 2014, 54% of the world’s population lived in urban areas. This trend is expected to continue. It is predicted that, by 2050, 66% of the entire population will be living in urban areas [1]. Lederbogen et al. [2] investigated the differences in the influence of stressors on the amygdala of urban and rural residents. In the social stress task, the activity of the amygdala, which is involved in stress processing, was higher in urban residents than in rural residents, which indicates that urban residents excessively react to stress [2]. “Technostress,” [3] caused by information and communication technology such as computers, smartphones, and social media also elevates contemporary stress [4]. In addition, Jiang et al. [5] reported that the attention enhancement benefits of green spaces were substantially counteracted using an electronic device in such settings. Increases in stressful situations in the modern society have drawn attention to the effects of exposure to natural stimuli that may promote relaxation [6].

Wood is a common natural material that has been used for building houses and furniture since ancient times. Empirically, it is known to have relaxation effects on humans and increasing data on its physiological effects on humans have been accumulated since 1992. According to a review conducted by Ikei et al. [7], 41 articles on the physiological relaxation effects conferred by wood to humans through the five senses (including 10 reviews and one book) were published between 1992 and 2016. Of these, 20, five, three, and two studies focused on the olfactory, visual, auditory, and tactile stimuli, respectively. This indicates that studies on the physiological effects of tactile stimuli conferred by wood in humans are limited.

Previously, we examined the physiological effects of touching the wood of Hinoki (Japanese cypress, *Chamaecyparis obtusa*) [8] and white oak (*Quercus alba*) [9,10] with the palm of the hand on the prefrontal cortex and autonomic nervous activities [8,9,10]. We observed that touching uncoated wood with the palm reduced prefrontal cortex activity [8,9,10], increased parasympathetic nervous activity [8,9,10], and decreased the heart rate [10], which induced a state of physiological relaxation in the participants. In addition, Morikawa et al. [11] reported that variation in systolic blood pressure when touching artificial materials is huge but touching Hinoki is small. Sakuragawa et al. [12] evaluated the effects of tactile stimulation with wood at different temperatures on human physiology. They reported that blood pressure did not change on touching wood such as Hinoki, Japanese cedar, or oak and did not increase even on touching cooled oak.

We believe it is necessary to clarify the physiological effects of touching wood with the soles of the feet because wood is often used as a flooring material. This is particularly applicable for Asian countries such as Japan where it is customary to walk barefoot indoors. A similar previous study conducted by Berger et al. [13] evaluated the psychological effects of touching parquet flooring typically used in Europe with the soles of the feet. Brushed multilayer parquet flooring with oil finish, multilayer parquet flooring with lacquer finish, and smooth laminate flooring were used to test tactile stimulation. A questionnaire survey was conducted and the study participants described the brushed flooring with oil finish as “warm, rough, and soft.” Subjective preference for this flooring was higher than that for flooring with lacquer or laminate finish, which was described as “cold, smooth, and hard.” However, no study has yet evaluated the physiological effects of touching natural wood with the soles of the feet.

Hinoki is a common coniferous tree in Japan. Its wood has long been used as building material for shrines and temples such as in the Horyuji Temple, which is one of the oldest wooden structures in Japan. Even today, Hinoki wood is used for building interiors such as floors and walls. The Japanese are likely to have frequent and daily contact with Hinoki since it is a familiar material and people’s interest in its possible physiological effects is increasing. It is valuable to clarify to producers of wood and other material floors the physiological effects of tactile stimulation using Hinoki wood applied to the soles of the feet.

Therefore, this study aimed to clarify the physiological effects of touching Hinoki with the soles of the feet. Marble, which is a natural, non-wood building material, was used as the control. We are conducting research based on the hypothesis that various natural stimuli derived from plants induce physiological relaxation of the human body. In this study, we hypothesized that contact with wood by the soles of the feet induces physiological relaxation in humans since physiological relaxation was observed when participants touched wood with the palms of their right hands in our previous research [8,9,10,11,12]. To evaluate physiological responses, we measured prefrontal cortex activity (in terms of oxy-hemoglobin [oxy-Hb] concentrations in the left and the right prefrontal cortices using near-infrared time-resolved spectroscopy [TRS]) and autonomic nervous activity (sympathetic and parasympathetic nervous activities assessed in terms of the heart rate variability [HRV] and the heart rate). To evaluate the psychological effects caused by contact with the materials, we used the modified semantic differential method. We compared physiological responses (1) between the two materials (i.e., Hinoki vs. marble) and (2) before and after touching each material (i.e., pre-measurement vs. post-measurement).

## 2. Materials and Methods

### 2.1. Participants

Totally, 19 Japanese female university-attending students (mean age, 21.2 ± 0.3 years) were included in this study. None of the participants reported any physiological or psychiatric disorders as well as any smoking habit in their personal histories. Furthermore, none of the participants were menstruating on the day of the experiment. All participants provided written informed consent for participation after being informed about the study’s aims and procedures. This study was conducted in accordance with the Declaration of Helsinki and under the regulations of the Ethics Committee of the Center for Environment, Health and Field Sciences, Chiba University, Japan (Project Identification Code Number 5).

### 2.2. Study Protocol

Physiological effects were measured in a chamber with an artificial climate maintained at 25 °C, 50% relative humidity, and 230-lux illumination.

**Figure 1:**
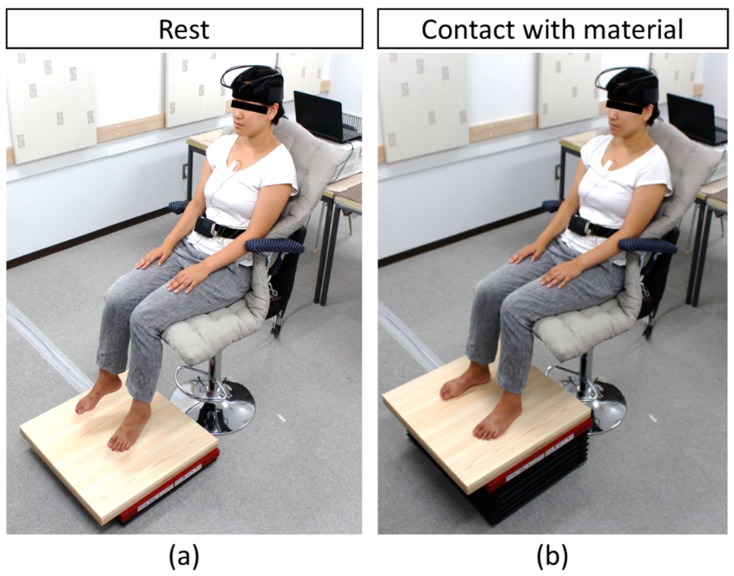
Experimental setup during rest (**a**) and tactile stimulation (**b**).

**Figure 2:**
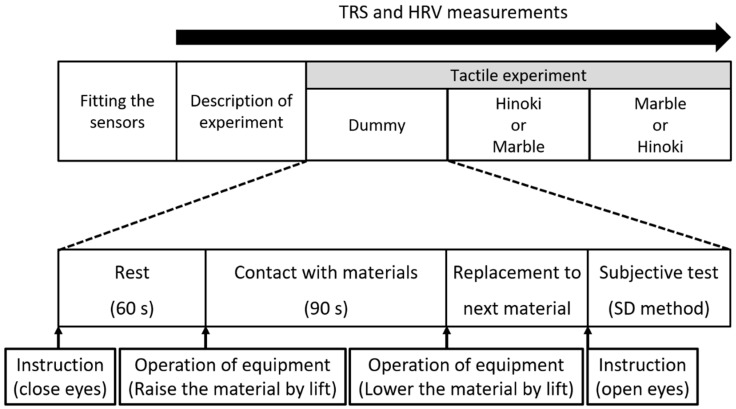
Procedure of the entire experiment. TRS, near-infrared time-resolved spectroscopy, HRV, heart rate variability, and SD, semantic differential.

Figure 1 presents the experimental setup while Figure 2 presents the experiment protocol. Sensors for physiological measurements were attached to each participant’s forehead and chest. After receiving information about the overall measurement flow, the participants practiced touching the sample with the soles of their feet by using a dummy material (plastic board) through the same procedure followed for the actual measurement. The participants rested with their eyes closed for 60 s (Figure 1a). By raising the sample using a scissor lift, the participants passively touched the samples with the soles of their feet while keeping their eyes closed for 90 s (Figure 1b). The contact was ended by lowering the sample with the scissor lift (Figure 1a). The experimenter then set another sample for the next measurement, laid a cloth on the sample, and told the participants to open their eyes. Next, subjective evaluation tests of the participants were performed. Stimulation with the subsequent materials was performed after a rest period of approximately 5 to 7 min. The materials were presented in a counterbalance order to eliminate any effects caused by the sequence of tactile stimulation.

### 2.3. Tactile Stimulation

**Figure 3:**
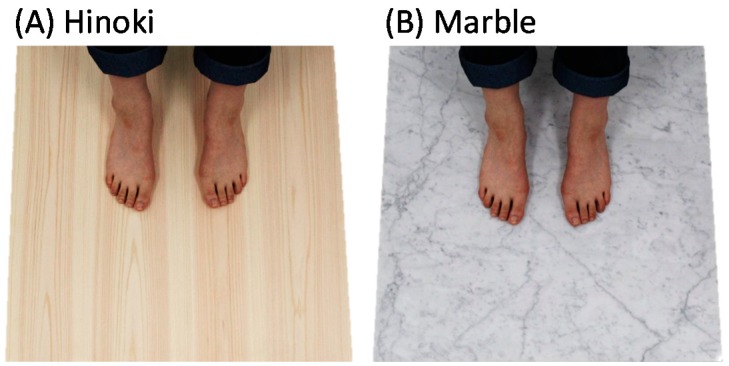
Materials used in the tactile experiment. (**A**) Uncoated Hinoki cypress wood and (**B**) marble.

Hinoki cypress (*Chamaecyparis obtusa*) wood grown in the Shizuoka Prefecture, Japan was used as the base material for the experiment. Four wooden laminae without vertical joints (600 × 150 × 20 mm) were bonded together along their width by using a water-based polymer isocyanate adhesive. To prevent bending, a second bonding was made using Japanese cedar (*Cryptomeria japonica*) plywood (600 × 600 × 25 mm). The total thickness of the resulting product was 45 mm. The surface of the Hinoki cypress material was finished by brushing it with a stainless-steel wire brush (Figure 3A). This wooden sample prepared for tactile stimulation has been hereafter referred to as “Hinoki.” A marble slab (600 × 600 × 20 mm) was selected as the control material. It was placed on a piece of cedar plywood of the same size as that of the plywood bonded to Hinoki. The surface was processed by buffing (Figure 3B). This marble sample has been, hereafter, referred to as “marble.” All materials were stored at room temperature. The physical properties of the materials are listed under Table 1.

### 2.4. Physiological Measurements

#### 2.4.1. Near-Infrared Time-Resolved Spectroscopy (TRS)

TRS was employed to assess prefrontal cortex activity. Sensors were mounted on the participant’s forehead and the oxy-Hb concentrations in the prefrontal cortex were measured using the TRS-20 system (Hamamatsu Photonics K.K., Shizuoka, Japan) [16,17,18]. Changes of blood flow (rCBF) associated with local nerve activity in the brain are correlated with changes of oxy-Hb and total Hb levels in near-infrared spectroscopy measurements [19,20,21]. Changes of the oxy-Hb concentration as measured by TRS reflect prefrontal activity because of sensor attachment to the forehead [16]. It has been reported that oxy-Hb concentrations in the prefrontal cortex are reduced by pleasant emotions and increased by unpleasant emotions [22]. The oxy-Hb concentrations in the left and right prefrontal cortices were measured for 30 s before (pre-measurement) and during the 90 s duration in which the participants’ soles touched the materials (post-measurement). All data were transformed by linear interpolation to every 1 s because their sampling intervals were approximately 1.07 to 1.11 s. In addition, post-measurement values (at every second) were calculated as the differences between the pre-measurement values (mean 30 s). In addition, the total hemoglobin (total Hb) and deoxygenated hemoglobin (deoxy-Hb) concentrations were measured and calculated as described for the oxy-Hb concentration.

#### 2.4.2. Heart Rate Variability (HRV) and Heart Rate

HRV and heart rate were employed as indicators of autonomic nervous activity. HRV was analyzed for the periods between consecutive R waves (R–R intervals, RRI), which is measured by a portable electrocardiograph (Activtracer AC-301A, GMS, Tokyo, Japan) [23,24]. This device uses a three-lead electrocardiogram (Lead II) to perform necessary measurements. Power levels of the high frequency (HF, 0.15–0.40 Hz) and low frequency (LF, 0.04–0.15 Hz) components of HRV were calculated using the maximum entropy method (MemCalc/Win, GMS, Tokyo, Japan) [25,26]. The HF power was considered to indicate the parasympathetic nervous activity while the LF/HF power ratio was considered to indicate the sympathetic nervous activity [27,28]. Here, the natural logarithmic values of the HF, i.e., ln(HF), power, and the LE/HF, i.e., ln(LF/HF), power ratios were employed to normalize the HRV parameters across participants [29]. The mean HRV and heart rate were calculated for the 90 s during which the participants’ soles were in contact with each material.

### 2.5. Psychological Measurements

Apart from the physiological measurements, a questionnaire was conducted to investigate psychological responses. The questionnaire was administered after touching each material. Evaluation using the modified semantic differential (SD) method [30] was performed using seven pairs of adjectives on 13 scales including “comfortable–uncomfortable”, “relaxed–awakening”, “natural–artificial”, “warm–cold”, “uneven–flat”, “dry–moist”, and “soft–hard”.

### 2.6. Statistical Analysis

The Statistical Package for the Social Sciences software (version 21.0, IBM Corp, Armonk, NY, USA) was used for all statistical analyses. In all cases, *p* < 0.05 was considered statistically significant.

Paired *t*-tests with the Holm correction were used to compare the oxy-Hb concentrations in the left and right prefrontal cortices occurring every 30 s between the Hinoki and marble materials. Thus, the Holm correction [31] was applied thrice. First, all *p* values were sorted, according to size, and then compared with their increasing limits: the smallest *p* value vs. 0.05/3, the second-smallest *p* value vs. 0.05/2, and the third-smallest *p* value vs. 0.05/1. To measure mean values over the 90-s contact period, paired *t*-tests were used to compare the physiological response. Wilcoxon signed-rank tests were applied to analyze differences in psychological indices between the two materials. 

We hypothesized that humans would be more relaxed by touching wood rather than other materials. Hence, we used one-sided tests for all comparisons.

## 3. Results

### 3.1. Physiological Effects

#### 3.1.1. TRS

**Figure 4:**
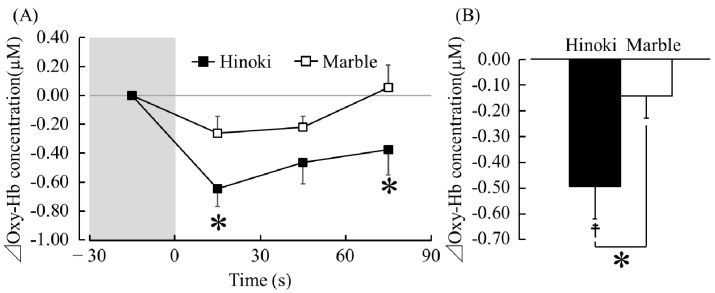
Oxy-Hb concentrations in the left prefrontal cortex during contact with materials via the soles of the feet. (**A**) Changes of the oxy-Hb concentrations. *n* = 19, mean ± standard error (SE), * *p* < 0.05 (Hinoki vs. marble) as determined using paired *t*-test with Holm correction. (**B**) Overall mean oxy-Hb concentrations. *n* = 19, mean ± SE, † *p* < 0.05 (pre- measurement vs. post-measurement value), * *p* < 0.05 (Hinoki vs. marble) as determined using a paired *t*-test.

Figure 4A presents the changes in the oxy-Hb concentrations that occurred in the left prefrontal cortex while the participants’ soles were in contact with Hinoki and marble. Between 1 s (immediately after touching) and 30 s (in contact with Hinoki, −0.65 ± 0.13 μM, in contact with marble, −0.26 ± 0.18 μM) and between 61 and 90 s (in contact with Hinoki, −0.37 ± 0.18 μM, in contact with marble, 0.05 ± 0.16 μM), the oxy-Hb concentrations significantly decreased during contact with Hinoki than with marble (Figure 4A, Holm corrected *p* < 0.05). Figure 4B presents a comparison of the average differential (post-measurement to pre-measurement) oxy-Hb concentrations in the left prefrontal cortex while the participants were in contact with Hinoki and marble. The average oxy-Hb concentrations in the left prefrontal cortex were −0.50 ± 0.13 µM during contact with Hinoki and −0.14 ± 0.09 µM during contact with marble. The oxy-Hb concentrations significantly decreased while touching Hinoki than while touching marble (Figure 4B, *p* < 0.05). Compared with the pre-measurement values, the oxy-Hb concentrations also significantly decreased after touching Hinoki (Figure 4B; *p* < 0.05). Incidentally, the mean baseline oxy-Hb concentrations in the 30 s before contact did not significantly differ between Hinoki and marble (Hinoki, 42.70 ± 1.34 µM, marble, 42.71 ± 1.36 µM, *p* > 0.05).

**Figure 5:**
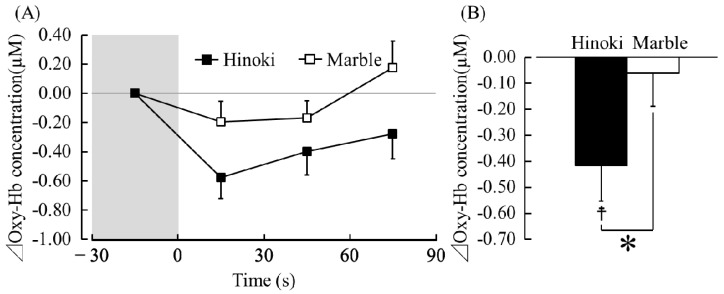
Oxy-Hb concentrations in the right prefrontal cortex during contact with materials via the soles of the feet. (**A**) Changes of the oxy-Hb concentration. *n* = 19, mean ± SE. (**B**) Overall mean oxy-Hb concentration. *n* = 19, mean ± SE, † *p* < 0.05 (pre-measurement vs. post-measurement value) * *p* < 0.05 (Hinoki vs. marble) as determined by using a paired *t*-test.

Figure 5A presents the changes in the oxy-Hb concentrations that occurred in the right prefrontal cortex while the participants’ soles were in contact with Hinoki and marble. Figure 5B presents a comparison of the average differential oxy-Hb concentrations in the right prefrontal cortex while the participants’ soles were in contact with Hinoki and marble. The average oxy-Hb concentrations in the right prefrontal cortex were −0.42 ± 0.14 µM during contact with Hinoki and −0.06 ± 0.13 µM during contact with marble. The oxy-Hb concentrations significantly decreased while touching Hinoki than while touching marble (Figure 5B, *p* < 0.05). Compared with the pre-measurement value, the oxy-Hb concentrations also significantly decreased after touching Hinoki (Figure 5B, *p* < 0.05). Incidentally, the mean baseline oxy-Hb concentrations during the 30 s before contact did not significantly differ between Hinoki and marble (Hinoki, 43.62 ± 1.24 µM, marble, 43.78 ± 1.34 µM, *p* > 0.05).

The average total Hb concentrations in the left prefrontal cortex were −0.48 ± 0.10 µM during contact with Hinoki and −0.27 ± 0.06 µM during contact with marble. The total Hb concentrations were significantly lower while touching Hinoki than while touching marble (*p* < 0.05). Compared with the pre-measurement values, the total Hb concentrations were also significantly lower after touching Hinoki (*p* < 0.05). Incidentally, the mean baseline of total Hb concentrations in the 30 s before contact did not significantly differ between Hinoki and marble (Hinoki, 65.53 ± 1.55 µM, marble, 65.61 ± 1.57 µM, *p* > 0.05). In the right prefrontal cortex, the total Hb concentrations were −0.51 ± 0.09 µM during contact with Hinoki and −0.25 ± 0.10 µM during contact with marble. The total Hb concentrations were significantly lower while touching Hinoki than while touching marble (*p* < 0.05). Compared with the pre-measurement values, the total Hb concentrations were also significantly lower after touching Hinoki (*p* < 0.05). Incidentally, the mean baseline total Hb concentrations in the 30 s before contact did not significantly differ between Hinoki and marble (Hinoki, 65.87 ± 1.47 µM, marble, 65.95 ± 1.40 µM, *p* > 0.05). As described previously, the total Hb concentrations in the left and right prefrontal cortices followed the same trend as the oxy-Hb concentration.

However, no differences were observed for the deoxy-Hb concentration between Hinoki and marble or between the pre-measurement and post-measurement values (Hinoki, 0.02 ± 0.05 µM, marble, −0.01 ± 0.05 µM, *p* > 0.05). The same results were recorded in the right prefrontal cortex (Hinoki, −0.05 ± 0.07 µM, marble, −0.08 ± 0.05 µM, *p* > 0.05).

#### 3.1.2. HRV and Heart Rate

**Figure 6:**
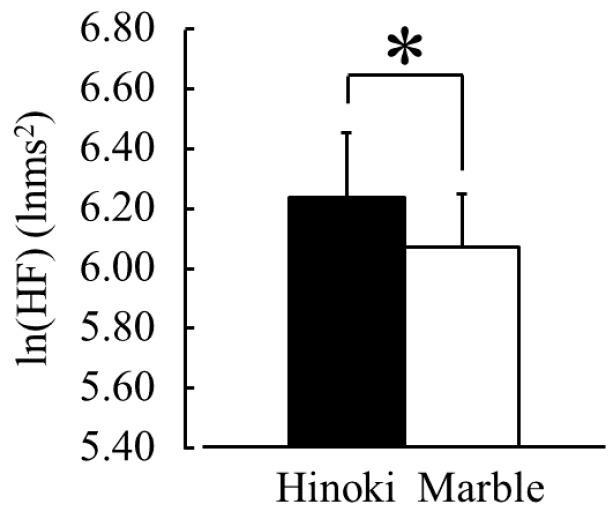
The natural logarithm of the high-frequency power component of heart rate variability during contact with materials via the soles of the feet. *n* = 19, mean ± SE, * *p* < 0.05 (Hinoki vs. marble) as determined by using a paired *t*-test.

Figure 6 presents a comparison of the changes of ln(HF), which indicates parasympathetic nervous activity, during contact with Hinoki and marble. The ln(HF) values were 6.24 ± 0.21 lnms^2^ during contact with Hinoki and 6.07 ± 0.18 lnms^2^ during contact with marble. The ln(HF) values significantly increased while touching Hinoki was compared to the findings from touching marble (Figure 6, *p* < 0.05). Incidentally, the mean baseline ln(HF) values during the 30 s before contact did not significantly differ between Hinoki and marble (6.06 ± 0.23 vs. 6.08 ± 0.21 lnms^2^, *p* > 0.05).

**Figure 7:**
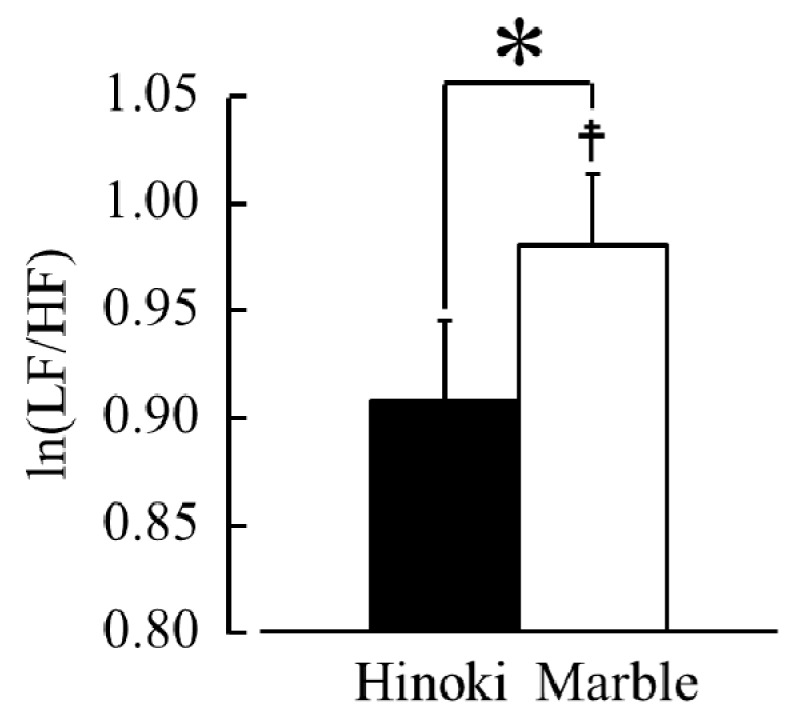
The natural logarithm of the low frequency/high frequency of heart rate variability during contact with materials via the soles of the feet. *n* = 19, mean ± SE, † *p* < 0.05 (pre-measurement vs. post-measurement value), * *p* < 0.05 (Hinoki vs. marble) as determined by using a paired *t*-test.

Figure 7 presents a comparison of the changes of the ln(LF/HF) ratios, which indicate sympathetic nervous activity, during contact with Hinoki and marble. The ln(LF/HF) ratios were 0.91 ± 0.04 while touching Hinoki and 0.98 ± 0.03 while touching marble. The ln(LF/HF) ratios were significantly lower while touching Hinoki than while touching marble (Figure 7, *p* < 0.05). Compared with the pre-measurement value, the ln(LF/HF) ratios significantly increased after touching marble (Figure 7, *p* < 0.05). Incidentally, the mean baseline ln(LF/HF) values during the 30 s before contact did not significantly differ between Hinoki and marble (0.90 ± 0.05 vs. 0.91 ± 0.04, *p* > 0.05).

However, no significant differences were found in the heart rate while touching Hinoki and marble (68.08 ± 1.49 vs. 67.92 ± 1.47 beats/min, *p* > 0.05).

### 3.2. Psychological Effects

**Figure 8:**
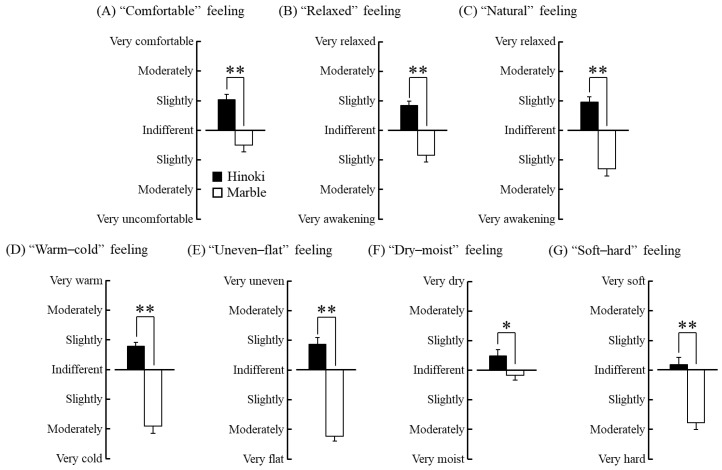
Results of the subjective evaluation using the modified semantic differential method after contact with materials via the soles of the feet. (**A**) “Comfortable” feeling. (**B**) “Relaxed” feeling, (**C**) “Natural” feeling, (**D**) “Warm–cold” feeling, (**E**) “Uneven–flat” feeling, (**F**) “Dry–moist” feeling, and (**G**) “Soft–hard” feeling. N = 19, mean ± SE, ** *p* < 0.01, * *p* < 0.05, as determined by using the Wilcoxon signed-rank test.

Results of the subjective evaluation using the modified SD method are presented in Figure 8. The participants subjectively reported feeling “slightly comfortable” after touching Hinoki and “indifferent to slightly uncomfortable” after touching marble. Therefore, touching Hinoki seemed to induce significantly more comfort than touching marble (Figure 8A, *p* < 0.01). Furthermore, the participants subjectively reported feeling “indifferent to slightly relaxed” while touching Hinoki and “indifferent to slight awakened” while touching marble. Thus, Hinoki appeared to have induced significantly more relaxation than marble (Figure 8B, *p* < 0.01). In addition, Hinoki was perceived as “slightly natural” and “significantly more natural” than marble, which was perceived as “slightly to moderately artificial” (Figure 8C, *p* < 0.01). The participants reported feeling “indifferent to slightly warm” after touching Hinoki and “slightly to moderately cold” after touching marble. Thus, Hinoki was perceived as being significantly warmer than marble (Figure 8D, *p* < 0.01). Furthermore, Hinoki was perceived as “indifferent to slightly uneven” and as “significantly more uneven than marble,” which was perceived as “moderate to very flat” (Figure 8E, *p* < 0.01). In addition, the participants reported feeling “indifferent to slightly dry” and “significantly drier than marble,” which was perceived as “indifferent to slightly moist” after touching Hinoki (Figure 8F, *p* < 0.05). Lastly, the patients reported feeling “indifferent to slightly soft” and “significantly softer than marble,” which was perceived as “slightly to moderately hard” after touching Hinoki (Figure 8G, *p* < 0.01).

## 4. Discussion

In this study, we clarified the effects of tactile stimulation with Hinoki with the soles of the feet on the prefrontal cortex activity using TRS and on the autonomic nervous activity using HRV. The overall mean values indicated that, as compared with touching the control (marble), touching Hinoki significantly decreased the oxy-Hb concentration in the left and right prefrontal cortices and significantly increased the ln(HF), which indicated increased parasympathetic nervous activity, and significantly decreased the ln(LF/HF) ratio, which indicated the sympathetic nervous activity. In addition, subjective evaluations demonstrated that the response to touching Hinoki was perceived as being more “comfortable”, “relaxed”, “natural”, “warm”, “uneven”, “dry”, and “soft”.

In this study, it was revealed that the oxy-Hb and total Hb concentrations of the prefrontal cortex were significantly decreased while touching Hinoki with the soles of the feet. Horiuchi et al. [32] report that the oxy-Hb and total Hb concentrations were decreased by visual stimulation using a forest landscape. Geroge et al. [33] reported that pleasant feelings cause a significant decrease in the cerebral blood flow. Similarly, Hoshi et al. [22] demonstrated that pleasant feelings decrease oxy-Hb levels and unpleasant feelings increase its levels in a screen image study. Therefore, the significant decreases of the oxy-Hb and total Hb concentrations during contact with Hinoki via the soles of the feet in this study might reflect the physiological relaxation effects of Hinoki wood.

Previously, we examined the physiological effects of tactile stimulation of the palm of the right hand with Hinoki on the prefrontal cortex and autonomic nervous activities [8]. As compared with marble, tactile stimulation with Hinoki significantly decreased the oxy-Hb concentration in the left prefrontal cortex and increased the ln(HF) power of HRV, which indicates increased parasympathetic nervous activity. We also examined the physiological effects of tactile stimulation with white oak, which is commonly used as a flooring material in comparison with other materials [9] and various coated woods [10]. We found that touching white oak with the palm of the right hand decreased the oxy-Hb concentration in the left and right prefrontal cortices [9,10] and increased ln(HF), which indicated increased parasympathetic nervous activity [9,10] and decreased the heart rate of the participants [11]. These physiological responses signify the induction of physiological relaxation in the participants. Thus, our findings, which are consistent with those of the previously mentioned studies, demonstrate a physiological relaxation effect of touching Hinoki with the soles of the feet.

Previous studies [34,35,36,37] have also demonstrated physiological effects of olfactory stimulation with Hinoki, which are consistent with the findings of the present study. In one such study, the physiological effects of olfactory stimulation were compared by using Hinoki wood chips that had been air-dried for 45 months and those that were rapidly dried at a high temperature using a drying machine [34]. Olfactory stimulation with air-dried chips rather than high-temperature-dried chips was found to reduce the prefrontal cortex activity [34]. Furthermore, olfactory stimulation with Hinoki leaf oil was shown to reduce the prefrontal cortex activity and increase the parasympathetic nervous activity, which highlights the physiological relaxation effects of olfactory stimulation with Hinoki leaf oil [35]. Inhalation of α-pinene [36] and D-limonene [37], which are two of the main odorous components of coniferous trees such as Hinoki, was found to increase parasympathetic nervous activity and decrease the heart rate, which indicates the induction of physiological relaxation.

Humans started evolving into their current form approximately 6 to 7 million years ago [38] and have spent more than 99.99% of our evolutionary history in the natural environment. Therefore, the human body is best adapted to a natural setting. The physiological function of humans is constantly stressed because they cannot cope with the sudden urbanization and artificial environment that developed after the Industrial Revolution. Humans, therefore, enter into a relaxed state when exposed to the natural environment or a nature-derived stimulation, which brings them closer to their original proper state as human beings [6,39,40]. We hypothesized that touching Hinoki with the soles of the feet induces physiological relaxation because wood shares closeness with humans and is a representative of natural materials. Previously, we had substantiated that natural environments or nature-derived stimuli (i.e., forests [41,42,43,44,45,46,47], urban parks [48,49,50,51], flowers [52,53,54,55,56], and foliage plants [57,58]) bring about physiological relaxation in humans. Wood is a common natural material that we often come in contact with in our daily life. When used as a flooring material, the main part of the human body that touches the wood is the soles of the feet and, as per the results of the present study, contact with wood can be effective in relieving stressful conditions in the modern society.

The novel characteristic of this study is the evaluation of the physiological effects of tactile stimulation, which is very limited in the literature. The study focused on the soles of the feet as the body contact points, which has not been evaluated in any previous study. The assessment involved simultaneous measurement of the prefrontal cortex and autonomic nervous activities to examine the possible relaxing effects of touching the material.

Altogether, our results reveal the physiological relaxation effects of touching Hinoki, which is a common coniferous tree in Japan, with the soles of the feet. However, this study has some limitations. First, it measured the physiological effects of tactile stimulation with wood with only the soles of the feet. In the future, it will be necessary to examine the physiological influence of active contact such as stroking with the foot or hand. Second, this study compared uncoated Hinoki with a non-wood material (marble). Future studies should determine the physiological effects of touching Hinoki with various types of coatings because most wooden objects used in everyday life are coated. In addition, the Hinoki and marble samples used in this experiment were cured at room temperature (25 °C), but the surface temperatures of both materials were not measured. The results in this experiment appear to have been influenced by the differences in the temperature of the materials. It is also necessary to consider such differences in the future. Lastly, the study population was comprised of only young healthy females in their 20s. In this experiment, participants who were menstruating on the day of the experiment were excluded. In the case of female participants, it is hoped that further consideration of the menstrual stage will be conducted in the future. In addition, to generalize these findings, further studies comprising larger and more diverse samples are required. For example, evaluating the differences in the effects of such materials between young males and females in their 20s as well as among various age groups is required. In addition, it would be valuable to examine the effects in people with high levels of stress in daily life such as patients who are physically disabled (e.g., with spinal cord injury), have a mental illness (e.g., depression), or are undergoing rehabilitation.

## 5. Conclusions

Our study revealed that, when compared with marble contact, contact with Hinoki via the soles of the feet significantly (1) decreased the oxy-Hb concentrations in the left and right prefrontal cortices, (2) increased parasympathetic nervous activity, and (3) decreased sympathetic nervous activity. These findings cumulatively indicate that tactile stimulation of the soles of the feet with Hinoki wood can effectively induce physiological relaxation effects.

## Figures and Tables

**Table 1:** The characteristics of the samples.

Material ^1^	*h* (mm)	*λ* [W/(m K)] ^2^	*ρ* (g/cm^3^) ^3^	*Ra* (µm) ^4^	Condition
Hinoki	20 (+JCP 25)	0.121	0.52	35.03	Brushing
Marble	20 (+JCP 25)	1.947	2.49	0.16	Buffing

*h*: Thickness of the material, *λ*: thermal conductivity, *ρ*: density of the material, *Ra*: arithmetic average roughness, and *JCP*: Japanese cedar plywood. ^1^ The samples were cut out from the center portion of the material (size, 300 × 300 mm) and their physical properties were assessed. Notably, Hinoki was used with the cedar plywood attached. ^2^ A heat flow meter (HFM 436 Lambda, NETZSCH, Selb, Germany) based on ASTM C518-10 [14] and ISO8310 [15] was used. Temperatures of the high-temperature and low-temperature heat plates were configured at 35 °C and 15 °C, respectively, and the direction of heat flow was set to vertically downward. Thermal conductivity was calculated at an average material temperature of 25 °C. ^3^ The mass was measured and the density was calculated. ^4^ A contact surface roughness profilometer (SE3500, Kosaka Laboratory Ltd., Tokyo, Japan) with a diamond stylus was used. The evaluation length was 50 mm and the cutoff value was 8 mm. The central portion of the samples were measured at five different time points with 50-mm spacing and the average value was calculated.
